# From 2-Alkylsulfanylimidazoles to 2-Alkylimidazoles: An Approach towards Metabolically More Stable p38α MAP Kinase Inhibitors

**DOI:** 10.3390/molecules22101729

**Published:** 2017-10-14

**Authors:** Fabian Heider, Urs Haun, Eva Döring, Mark Kudolo, Catharina Sessler, Wolfgang Albrecht, Stefan Laufer, Pierre Koch

**Affiliations:** 1 Department of Pharmaceutical and Medicinal Chemistry, Institute of Pharmaceutical Sciences, Eberhard Karls Universität Tübingen, Auf der Morgenstelle 8, 72076 Tübingen, Germany; fabian.heider@uni-tuebingen.de (F.H.); urs.haun@student.uni-tuebingen.de (U.H.); eva.doering@uni-tuebingen.de (E.D.); mark.kudolo@uni-tuebingen.de (M.K.); catharina.sessler@uni-tuebingen.de (C.S.); stefan.laufer@uni-tuebingen.de (S.L.); 2 Teva-ratiopharm, Graf-Arco-Str. 3, 89079 Ulm, Germany; wolfgang.albrecht@ratiopharm.de

**Keywords:** kinase inhibitors, p38α MAP kinase, trisubstituted imidazoles, metabolic stability, Alzheimer’s disease, neurodegenerative diseases, cancer

## Abstract

In vitro and in vivo metabolism studies revealed that 2-alkylsulfanylimidazole **ML3403** (4-(5-(4-fluorophenyl)-2-(methylthio)-1*H*-imidazol-4-yl)-*N*-(1-phenylethyl)pyridin-2-amine) undergoes rapid oxidation to the sulfoxide. Replacing the sulfur atom present in the two potent p38α mitogen-activated protein (MAP) kinase inhibitors **ML3403** and **LN950** (2-((5-(4-fluorophenyl)-4-(2-((3-methylbutan-2-yl)amino)pyridin-4-yl)-1*H*-imidazol-2-yl)thio)ethan-1-ol) by a methylene group resulted in 2-alkylimidazole derivatives **1** and **2**, respectively, having a remarkably improved metabolic stability. The 2-alkylimidazole analogs **1** and **2** showed 20% and 10% biotransformation after 4 h of incubation with human liver microsomes, respectively. They display a 4-fold increased binding affinity towards the target kinase as well as similar in vitro potency and ex vivo efficacy relative to their 2-alkylsulfanylimidazole counterparts **ML3403** and **LN950**. For example, 2-alkylimidazole **2**, the analog of **LN950**, inhibits both the p38α MAP kinase as well as the LPS-stimulated tumor necrosis factor-α release from human whole blood in the low double-digit nanomolar range.

## 1. Introduction

The p38α mitogen-activated protein (MAP) kinase is a ubiquitously expressed serine/threonine kinase, which is implicated in various cellular processes such as cell survival, proliferation and differentiation. Because it promotes the expression of pro-inflammatory cytokines such as tumor necrosis factor-α (TNF-α) and interleukin-1β (IL-1β), the p38α MAP kinase has received a lot of attention as a target for drug discovery programs since the mid-1990s. Several p38α MAP kinase inhibitors were tested in clinical trials against cancer and chronic inflammatory diseases like rheumatoid arthritis or chronic obstructive pulmonary disease. Despite these major efforts, up to date there is still no p38α MAP kinase inhibitor on the market, as most of the trials were terminated due to adverse events or lack of efficacy. However, recent studies suggest an important role of the kinase in the pathogenesis of neurodegenerative diseases like multiple sclerosis and Alzheimer’s disease [1,2,3]. Recently, two phase II studies of selective p38α MAP kinase inhibitor **VX-745** (Figure S1, supplementary materials) to treat Alzheimer’s disease have been completed [4,5].

**Figure 1:**
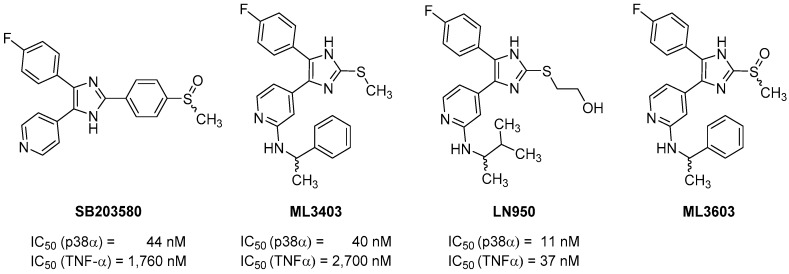
Pyridinylimidazoles as p38α MAP kinase inhibitors.

Developed by SmithKline Beecham Pharmaceuticals, **SB203580** is a trisubstituted imidazole representing one of the first prototypical p38α MAP kinase inhibitors (Figure 1). 

In collaboration with the University of Tübingen, Merckle GmbH disclosed a structural analog **ML3403**, which has been widely investigated in a variety of studies [6,7,8,9,10]. A further optimization study resulted in 2-(2-hydroxyethylsulfanyl)-4-(4-fluorophenyl)-5-(2-aminopyridin-4-yl)imidazole (**LN950**) [11]. Compared to **ML3403**, **LN950** displays an improved p38α MAP kinase inhibitory activity as well as a two orders of magnitude higher inhibition of lipopolysaccharide (LPS)-stimulated TNF-α release from human whole blood (HWB) (Figure 1).

Both inhibitors, **ML3403** and **LN950**, possess an alkylsulfanyl moiety in the imidazole-C2 position, which is prone to oxidation by metabolic enzymes such as cytochrome p450 (CYP). The metabolic stability of **ML3403** was extensively investigated in in vitro (animal and human liver microsomes) as well as in vivo (Wistar rats) studies [12,13]. Sulfoxide **ML3603** (Figure 1) was identified as the main metabolite in all studies and acts as an active metabolite. The conversion of **ML3403** to **ML3603** is mainly driven by the four CYP isoenzymes CYP1A2, CYP2C19, CYP2D6, and CYP3A4. The sulfoxide is then to a certain extent further metabolized to the corresponding sulfone; in addition *N*-dealkylation of the phenylethyl moiety as well as *N*-oxidation of the pyridine have also been observed. In phase II metabolism, *N*-conjugation of the dealkylated product by *N*-methyltransferase was predominantly registered. Kammerer et al. [13] showed the in vitro half-life of **ML3403** in male and female human liver microsomes (HLM) to be 32.7 min. In vitro experiments using mouse and rat liver microsomes also showed short half-lives of 5.9 and 11.4 min, respectively.

While the metabolite **ML3603** itself is active on p38α MAP kinase, it would be favorable to have a metabolically stable compound as a pharmacological tool compound since active metabolites can affect e.g., dosing calculations, dose-response relations and off-target effects.

**Figure 2:**
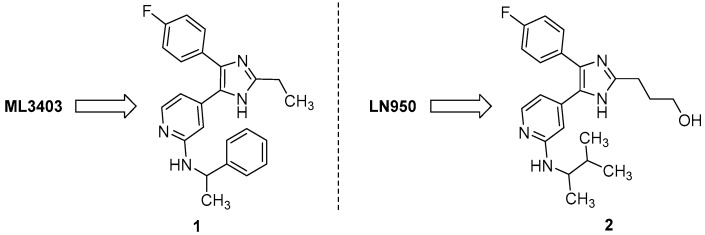
Modifications of **ML3403** and **LN950**.

In order to develop metabolically more stable p38α MAP kinase inhibitors, we removed the metabolic hot spot in **ML3403** and **LN950** and synthesized analogs of both inhibitors (compounds **1** and **2**), wherein the sulfur atom was replaced by a methylene group (Figure 2).

We evaluated the binding affinity as well as the inhibitory activity of the novel compounds toward the target kinase and their ability to inhibit the LPS-stimulated TNF-α release in human whole blood. The obtained biological data of **1** and **2** were compared to those of the parent compounds in order to estimate the influence of the sulfur atom present in **ML3403** and **LN950**. Moreover, the metabolic stability in human liver microsomes (HLM) of all four inhibitors was investigated. To rule out any interference and possible side-effects due to CYP inhibition, we subjected the compounds to a CYP inhibition assay.

## 2. Results and Discussion

### 2.1. Chemistry

**Scheme 1:**
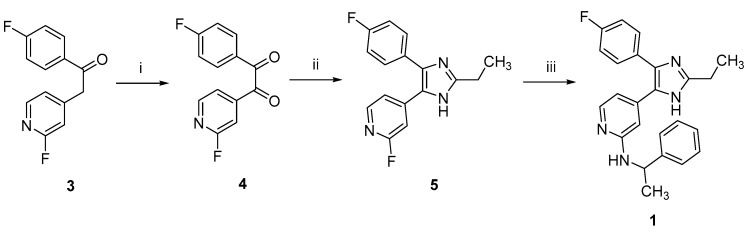
Synthesis of 2-ethylimidazole **1**. (i) SeO_2_, acetic acid, 50 °C, 3 h, 62%; (ii) propanal, 7 M ammonia in methanol, reflux temperature, 4 h, 43%; (iii) 1-phenylethylamine (excess), 160 °C, 72 h, 68%.

The synthesis of the **ML3403** analog **1** was performed in a concise three-step synthetic route, as depicted in Scheme 1. Ethanone **3** was oxidized to the corresponding α-diketone **4** in a Riley oxidation using selenium dioxide. The imidazole ring formation was achieved in a Radziszewski imidazole synthesis by reacting dione **4** in the presence of 7 M ammonia in methanol and propanal. In the last step, the amino moiety at the pyridine-C2 position was introduced via a nucleophilic aromatic substitution reaction.

**Scheme 2:**
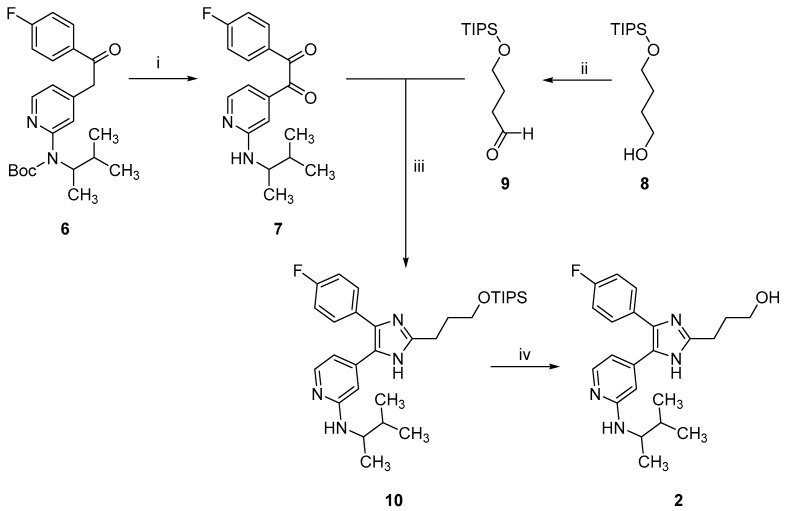
Synthesis of 2-(3-hydroxypropyl)imidazole **2**. (i) SeO_2_, acetic acid, reflux temperature, 1.5 h, 39%; (ii) *N*-methylimidazole, 2,2′-bipyridinyl, Cu(MeCN)_4_CF_3_SO_3_, (2,2,6,6-tetramethylpiperidin-1-yl)oxyl, MeCN, room temperature (rt), 1.5 h, 20%; (iii) NH_4_OAc, methanol, reflux temperature, 4 h, 20%; (iv) HCl, methanol, rt, 1 h, 73%.

For the synthesis of the **LN950** analog **2**, a similar strategy consisting of Riley oxidation of an ethanone followed by Radziszewski imidazole synthesis was pursued (Scheme 2). Ethanone **6** [11] already bears the 3-methylbut-2-ylamine moiety at the pyridine-C2 position as well as a Boc-protecting group. Riley oxidation of ethanone **6** under acidic conditions resulted in the α-diketone **7**, wherein the Boc-protecting group was cleaved, too.

Triisopropylsilyl (TIPS)-protected aldehyde **9** was synthesized by the oxidation of primary alcohol **8** using a copper(I)/(2,2,6,6-tetramethylpiperidin-1-yl)oxyl (TEMPO) catalyst system according to Hoover and Stahl [14]. The ring closing reaction of dione **7** and aldehyde **9** in the presence of ammonium acetate in methanol afforded imidazole **10**. Finally, the TIPS-protecting group was removed under acidic conditions to yield the trisubstituted imidazole **2**.

### 2.2. Biological Evaluation

2-Alkylsulfanyl imidazoles **1** and **2** were evaluated in an enzyme-linked immunosorbent assay (ELISA) [15] as well as in a fluorescence polarization (FP)-based assay [16] for their ability to inhibit and bind to the p38α MAP kinase. Moreover, the ability of the novel compounds to inhibit the LPS-stimulated TNF-α release in HWB was tested [17]. In this ex vivo assay, the efficacy of the inhibitors is determined more specifically with regard to pharmacokinetic characteristics such as cellular permeability and plasma protein binding. The obtained data of **1** and **2** were compared to those of their parent compounds **ML3403** and **LN950**, respectively. The results are listed in Table 1.

2-Alkylimidazoles **1** and **2** are both potent inhibitors and potent binders of the target kinase, displaying IC_50_ and K_i_ values down to the low single-digit nanomolar range.

Comparison of the biological data of the 2-alkylsulfanylimidazole derivatives **ML3403** and **LN950** with their 2-alkylimidazole counterparts **1** and **2** reveals the replacement of the sulfur atom by a methylene group not to have any influence on the biological activity of these inhibitors. However, the 2-alkylimidazole derivatives display a 4-fold stronger binding affinity toward p38α MAP kinase compared to their corresponding 2-alkylsulfanylimidazoles. The most potent 2-alkylimidazole derived inhibitor **2** is 3.6-fold more active than the p38α MAP kinase reference compound **ML3403**.

### 2.3. Microsomal Stability Studies

Inhibitors **ML3403**, **LN950**, **1** and **2** were further tested for their metabolic stability in pooled adult male & female HLM. 

**Figure 3:**
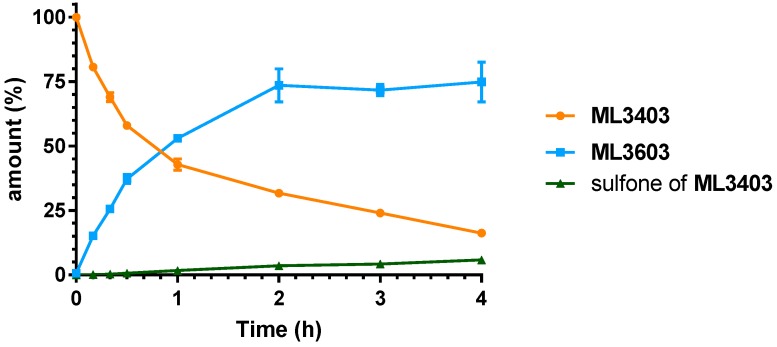
Metabolic stability of **ML3403** and the formation of main metabolites (sulfoxide **ML3603** and sulfone).

**ML3403** is rapidly metabolized in our study displaying an in vitro half-life of 0.86 h (Figure 3). After an incubation time of 4 h, less than 20% of **ML3403** were present (Figure 3 and Table S1, supplementary materials). Analysis of the metabolites by LC-MS (Tables S2–S5, supplementary materials) confirmed the aforementioned sulfoxide **ML3603** (*m*/*z* 421.5) to represent the main metabolite (almost 75% of all metabolites after 4 h). Up to 6% of the corresponding sulfone (*m*/*z* 437.4) was observed. Other low abundance metabolites were detected having an *m*/*z* ratio of 301.4 (*N*-dealkylation) and 317.5 (*N*-dealkylation + sulfoxidation). These findings are in agreement with the previously reported study by Kammerer et al. [13]. 

**Figure 4:**
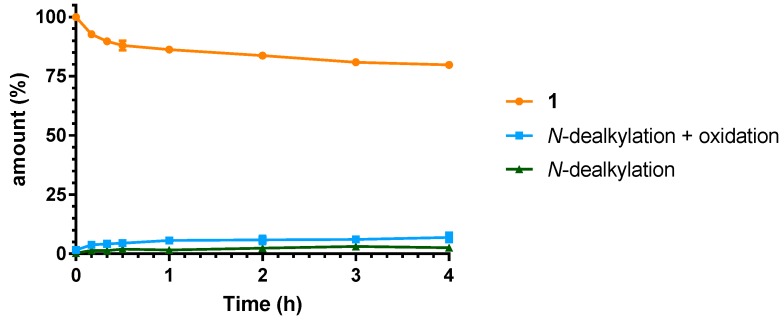
Metabolic stability of **1** and the formation of main metabolites (*N*-dealkylation as well as oxidation and *N*-dealkylation of **1**).

In contrast, 2-alkylimidazole **1** displays excellent metabolic stability remaining unmetabolized at up to 80% after the incubation time of 4 h (Figure 4 and Table S6, supplementary materials). Among the identified metabolites (Tables S7–S10, supplementary materials), *m*/*z* 283.6 was present, representing the *N*-dealkylated metabolite. Additionally, the LC-MS analysis shows two peaks in close proximity, both having an *m*/*z* ratio of 403.3. It is conceivable that both peaks correspond to the diastereomers resulting from the hydroxylation of the methylene group present in the ethyl moiety. The most prominent metabolite after the final sampling is seen at *m*/*z* 299.5 and is a combination of both *N*-dealkylation and possible hydroxylation, accounting for almost 50% of all arising metabolites.

**Figure 5:**
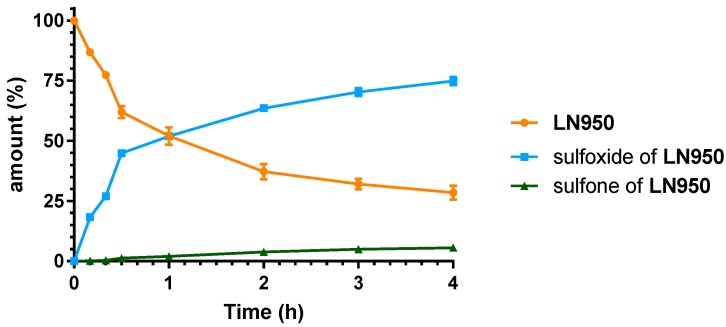
Metabolic stability of **LN950** and the formation of main metabolites (sulfoxide and sulfone of **LN950**).

**LN950** undergoes a similar but slightly slower biotransformation like **ML3403**. Over a time span of 4 h, **LN950** exhibits >70% degradation, most likely leading to the oxidation of the sulfur atom at the imidazole-C2 position (Figure 5 and Table S11, supplementary materials). Examination of the metabolites showed an almost identical pattern in comparison with **ML3403**, giving the corresponding sulfoxide as the main metabolite, as well as the sulfone and some *N*-dealkylated product (Tables S12–S14, supplementary materials). 

**Figure 6:**
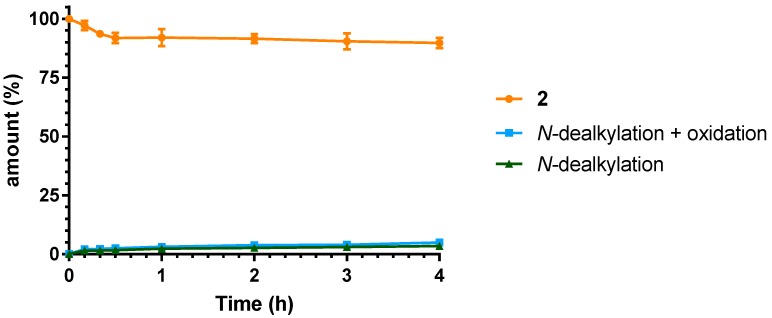
Metabolic stability of **2** and the formation of main metabolites (*N*-dealkylation as well as oxidation and *N*-dealkylation of **2**).

The **LN950** analog, imidazole **2**, wherein the sulfur atom was replaced by a methylene group, shows under same conditions an exquisite metabolic profile. After 4 h, a degradation of only 10% could be observed (Figure 6 and Table S15, supplementary materials). Several metabolites were detected, all in low quantities (Tables S16 and S17, supplementary materials). Overall, a similar metabolite pattern as that for compound **1** was observed. In detail, CYP-mediated oxidation (hydroxylation or *N*-oxide) and to a small extent *N*-dealkylation occured. Comparison of the substituents at the imidazole-C2 position of 2-alkylimidazoles **1** and **2** leads to the assumption that the more polar 2-hydroxyethyl moiety of **2** is less susceptible for hydroxylation by CYP than the ethyl moiety present in **1** when incubated with HLM. 

### 2.4. CYP Inhibition

Pyridinylimidazoles, like **SB203580**, are known to inhibit CYP isoenzymes due to the capability of the heterocyclic rings’ nitrogen atoms to coordinate with the iron present in these proteins [18]. High CYP inhibition is associated with side-effects such as hepatotoxicity and is also responsible for many clinically relevant drug-drug interactions [19]. 

In an initial screening, **ML3403** and **LN950** as well as imidazoles **1** and **2** were studied for the purpose of assessing their inhibitory activity on the CYP isoforms 1A2, 2C9, 2C19, 2D6 and 3A4 (Table 2). At a test concentration of 10 µM, **ML3403** displays more than 50% inhibition of four out of the five major drug metabolizing CYP isoforms. Compared to **ML3403**, **LN950** shows a reduced CYP inhibition profile. **LN950** is a low inhibitor of CYP isoenzymes 1A2 and 2D6 and exhibits moderate to high affinity for the other three tested CYP isoforms. The 2-alkylimidazoles **1** and **2** display a higher affinity toward the tested CYP isoforms compared to their corresponding 2-alkylsulfanylimidazole counterparts. 2-Alkylimidazoles **1** and **2** inhibit all five CYP isoforms by more than 70% and 60%, respectively. The most potent inhibitor, 2-alkylimidazole **2**, shows a similar inhibition of CYP isoforms 1A2 and 2C19 as well as a reduced 2C9 CYP inhibition in comparison to the p38α MAP kinase reference compound **ML3403**. However, the CYP isoforms 2D6 and 3A4 are inhibited more strongly by 2-alkylimidazole **2** than by **ML3403**. Since the tested concentration of 10 µM in the CYP inhibition assay represents almost 1000-fold the IC_50_ value of imidazole **2** in the kinase activity assay, a certain margin of safety might be given concerning CYP inhibition-associated side effects.

## 3. Materials and Methods 

### 3.1. Chemistry

Reagents and solvents were obtained from commercial sources and used without further purification. Thin layer chromatography (TLC) reaction controls were performed for all reactions using fluorescent silica gel 60 F_254_ plates (Merck, Darmstadt, Germany) and visualized under natural light and UV illumination at 254 and 366 nm. The purity of tested compounds **1** and **2** were determined by reverse phase high performance liquid chromatography (HPLC) (Agilent Technologies, Santa Clara, CA, USA). HPLC was carried out on an Agilent 1100 Series HPLC system, equipped with an UV DAD (detection at 218 nm, 254 nm and 280 nm). The chromatographic separation was performed on an XBridge^TM^ C18 column (Waters, Milford, MA, USA) (150 mm × 4.6 mm, 5 µm) at 30 °C oven temperature. The injection volume was 10 μL and the flow rate was 1.5 mL/min using the following gradient: 0.01 M KH_2_PO_4_, pH 2.3 (solvent A), methanol (solvent B), 45% B to 85% B in 9 min; 85% B for 6 min; stop time 16 min. Flash column chromatography was performed using an Interchim PuriFlash 430 automated flash chromatography system (Interchim, Montluçon, France) with Davisil LC60A 20–45 µm silica from Grace Davison or PuriFlash SIHP 30 µm columns. Nuclear magnetic resonance (NMR) spectra were measured on a Bruker Avance III HD NMR spectrometer (Bruker Daltonik GmbH, Bremen, Germany) at 300 MHz in the Organic Chemistry Institute, Eberhard Karls Universität Tübingen. The chemical shifts δ are reported in parts per million (ppm) relative to tetramethylsilane. All spectra were calibrated against the (residual proton) peak of the deuterated solvent used. Mass spectra were performed on an Advion Expression S electrospray ionization mass spectrometer (ESI-MS) with an Advion Plate Express (TLC interface) (Advion, Ithaka, NY, USA).

#### 3.1.1. Synthesis of 2-Alkylimidzole **1**

*1-(4-Fluorophenyl)-2-(2-fluoropyridin-4-yl)ethane-1,2-dione* (**4**) [20]. 1-(4-Fluorophenyl)-2-(2-fluoropyridin-4-yl)ethan-1-one (**3**) (1500 mg, 6.43 mmol) was dissolved in glacial acetic acid (10 mL) and selenium dioxide (928 mg, 8.36 mmol) was added. The reaction mixture was heated to 50 °C for 3 h. After cooling to rt, the reaction was filtered and the solvent was removed under reduced pressure. The crude product was taken up in ethyl acetate and washed with saturated NaHCO_3_ solution. The aqueous layer was adjusted to pH 10 with 1 M aq. NaOH solution and extracted twice with ethyl acetate. The combined organic layers were dried over anhydrous Na_2_SO_4_, the solvent was removed under reduced pressure and the residue was purified by flash chromatography (SiO_2_, *n*-hexane/EtOAc 9:1 to 7:3) to give an orange solid (987 mg, 62%). ^1^H-NMR (300 MHz, DMSO-*d*_6_) δ 7.43–7.53 (m, 2H), 7.69 (d, *J* = 0.8 Hz, 1H), 7.83 (dt, *J* = 5.0, 1.7 Hz, 1H), 8.09–8.18 (m, 2H), 8.55 (d, *J* = 5.1 Hz, 1H); MS-ESI (*m*/*z*) 301.9 [M + Na + MeOH]^+^; HPLC: t_R_ = 5.73 min.

*4-(2-Ethyl-4-(4-fluorophenyl)-1H-imidazol-5-yl)-2-fluoropyridine* (**5**). Compound **4** (250 mg, 1.01 mmol) was dissolved in methanol (5 mL). Subsequently, 7 M ammonia in methanol solution (2.89 mL, 20.23 mmol) and propionaldehyde (88.11 mg, 1.52 mmol) were added successively. The reaction mixture was heated to 80 °C for 4 h. After removing the solvent under reduced pressure, the crude product was directly purified by flash chromatography (SiO_2_, DCM/EtOH 97:3) to give a white solid (125 mg, 43%). ^1^H-NMR (300 MHz, DMSO-*d*_6_) δ 1.28 (t, *J* = 7.6 Hz, 3H), 2.64–2.77 (m, 2H), 7.09 (s, 1H), 7.17–7.40 (m, 3H), 7.47–7.56 (m, 2H), 8.06 (d, *J* = 5.4 Hz, 1H), 12.41 (br. s, 1H); MS-ESI (*m*/*z*) 286.0 [M + H]^+^, 284.0 [M − H]^−^; HPLC: t_R_ = 3.68 min. 

*4-(2-Ethyl-4-(4-fluorophenyl)-1H-imidazol-5-yl)-N-(1-phenylethyl)pyridin-2-amine* (**1**). Compound **5** (60 mg, 0.21 mmol) was dissolved in α-methylbenzylamine (1 mL) and heated for 72 h to 160 °C. After cooling to rt, the crude mixture was purified by flash chromatography (SiO_2_, DCM/EtOH 97:3) to give a white solid (57 mg, 68%). ^1^H-NMR (300 MHz, CDCl_3_) δ 1.18–1.25 (m, 3H), 1.38–1.44 (m, 3H), 2.63 (q, *J* = 7.6 Hz, 2H), 4.44 (quin, *J* = 6.5 Hz, 1H), 5.03 (d, *J* = 5.7 Hz, 1H), 6.3 (br. s, 1H), 6.65 (d, *J* = 4.7 Hz, 1H), 6.91–6.99 (m, 2H), 7.10–7.25 (m, 5H), 7.33 (dd, *J* = 8.0, 5.6 Hz, 2H), 7.90 (d, *J* = 5.4 Hz, 1H); ^13^C-NMR (75 MHz, CDCl_3_) δ 12.6, 21.7, 24.3, 51.8, 104.0, 111.4, 115.5 (d, *J* = 21.6 Hz), 125.6, 126.9, 128.5, 130.0 (d, *J* = 8.3 Hz), 144.3, 147.8, 150.2, 158.1, 162.3 (d, *J* = 247.7 Hz); MS-ESI (*m*/*z*) 387.1 [M + H]^+^; 385.1 [M − H]^−^; HPLC: t_R_ = 7.23 min (100% purity).

#### 3.1.2. Synthesis of 2-Alkylimidzole **2**

*4-(Triisopropylsilyl)oxy)butanal* (**9**). 4-(Triisopropylsilyl)oxy)butan-1-ol (**8**) [21] (4,600 mg, 18.68 mmol) was dissolved in MeCN (50 mL) before Cu(MeCN)_4_CF_3_SO_3_ (352 mg, 0.93 mmol), 2,2′-bipyridyl (149 mg, 0.93 mmol), TEMPO (146 mg, 0.93 mmol) and *N*-methylimidazole (154 mg, 1.87 mmol) were added successively. The mixture was stirred at rt for 16 h. *n*-Hexane and water were added and the organic layer was separated. The aqueous layer was extracted three times with diethyl ether. The combined organic layers were washed with brine and dried over anhydrous Na_2_SO_4_. The solvent was removed under reduced pressure and the residue was purified by flash chromatography (SiO_2_, *n*-hexane/EtOAc 90:10) to yield a yellowish oil (2580 mg, 56%). ^1^H-NMR (300 MHz, CDCl_3_) δ 0.99–1.13 (m, 21H), 1.67–1.79 (m, 2H), 2.55 (m, 2H), 3.72 (dt, *J* = 16.6, 6.5 Hz, 2H), 9.39 (s, 1H).

*1-(4-Fluorophenyl)-2-(2-((3-methylbutan-2-yl)amino)pyridin-4-yl)ethane-1,2-dione* (**7**). *tert*-Butyl-(4-(2-(4-fluorophenyl)-2-oxoethyl)pyridin-2-yl)(3-methylbutan-2-yl)carbamate (**6**) (395 mg, 0.99 mmol) was dissolved in glacial acetic acid (8 mL). Selenium dioxide (142 mg, 1.28 mmol) was added and the reaction was heated to reflux temperature for 1.5 h. After cooling to rt, the reaction was filtered and the solvent was removed under reduced pressure. The crude product was taken up in ethyl acetate and washed with saturated NaHCO_3_ solution. The aqueous layer was adjusted to pH 10 with 1 M aq. NaOH and extracted twice with ethyl acetate. The combined organic layers were dried over anhydrous Na_2_SO_4_, the solvent was removed under reduced pressure and the residue was purified by flash chromatography (SiO_2_, *n*-hexane/EtOAc 80:20 to 60:40) to give a white solid (121 mg, 39%). ^1^H-NMR (300 MHz, CDCl_3_) δ 0.95 (dd, *J* = 10.0, 6.8 Hz, 6H), 1.15 (d, *J* = 6.5 Hz, 3H), 1.82 (dq, *J* = 12.3, 6.8 Hz, 1H), 3.69–3.78 (m, 1H), 6.82 (s, 1H), 6.91 (dd, *J* = 5.2, 1.4 Hz, 1H), 7.18–7.25 (m, 2H), 7.94–8.09 (m, 2H), 8.25 (dd, *J* = 5.2, 0.5 Hz, 1H); MS-ESI (*m*/*z*) 315.1 [M + H]^+^; 313.1 [M − H]^−^; HPLC: t_R_ = 5.07 min.

*4-(4-(4-Fluorophenyl)-2-(3-((triisopropylsilyl)oxy)propyl)-1H-imidazol-5-yl)-N-(3-methylbutan-2-yl)pyridin-2-amine* (**10**). Compound **7** (125 mg, 0.40 mmol) was dissolved in methanol (10 mL). Ammonium acetate (613 mg, 7.95 mmol) and aldehyde **9** (147 mg, 0.60 mmol) were added. The reaction was heated to 80 °C for 4 h. After cooling to rt, saturated NaHCO_3_ solution was added. The aqueous phase was adjusted to pH 10 with 1 M aq. NaOH and extracted thrice with ethyl acetate. The combined organic layers were dried over anhydrous Na_2_SO_4_, the solvent was removed under reduced pressure and the residue was purified by flash chromatography (SiO_2_, *n*-hexane/EtOAc 50:50 to 0:100) to give a colorless oil (43 mg, 20%). ^1^H-NMR (300 MHz, CDCl_3_) δ 0.87 (dd, *J* = 6.8, 1.6 Hz, 6H), 1.03–1.05 (m, 24H), 1.71 (td, *J* = 6.7, 5.4 Hz, 1H), 1.93–2.06 (m, 2H), 2.96 (d, *J* = 6.1 Hz, 2H), 3.34–3.51 (m, 1H), 3.84 (d, *J* = 4.5 Hz, 2H), 4.39 (d, *J* = 8.2 Hz, 1H), 6.19–6.54 (m, 1H), 6.57–6.70 (m, 1H), 6.94–7.15 (m, 2H), 7.31–7.44 (m, 1H), 7.57 (br. s, 1H), 7.89 (d, *J* = 4.9 Hz, 1H), 9.96–10.24 (m, 1H); MS-ESI (*m*/*z*) 539.2 [M + H]^+^, 537.1 [M − H]^−^; HPLC: t_R_ = 5.12 min.

*3-(4-(4-Fluorophenyl)-5-(2-((3-methylbutan-2-yl)amino)pyridin-4-yl)-1H-imidazol-2-yl)propan-1-ol* (**2**). Compound **10** (125 mg, 0.40 mmol) was dissolved in methanol (10 mL) and 2 M aq. HCl solution was added. The reaction was stirred at rt for 1 h. The organic solvent was removed under reduced pressure and the aqueous residue was treated with saturated NaHCO_3_ solution. A white precipitate was formed, collected by filtration and taken up in ethyl acetate. The organic solution was dried over anhydrous Na_2_SO_4_ and the compound was purified by flash chromatography (SiO_2_, DCM/EtOH 92:08 to 85:15) to give an off-white solid (22 mg, 73%). ^1^H-NMR (300 MHz, CDCl_3_) δ 0.85 (d, *J* = 6.5 Hz, 6H), 1.02 (d, *J* = 6.6 Hz, 3H), 1.63–1.75 (m, 1H), 1.98 (dt, *J* = 11.5, 5.8 Hz, 2H), 2.86–2.96 (m, 2H), 3.32–3.43 (m, 1H), 3.79 (t, *J* = 5.3 Hz, 2H), 4.53 (d, *J* = 8.5 Hz, 1H), 6.45 (s, 1H), 6.57 (d, *J* = 4.5 Hz, 1H), 7.03 (t, *J* = 8.6 Hz, 2H), 7.46 (dd, *J* = 8.6, 5.4 Hz, 2H), 7.90 (d, *J* = 5.4 Hz, 1H); ^13^C-NMR (75 MHz, DMSO-*d_6_*) δ 16.9, 17.7, 18.8, 26.5, 29.8, 32.5, 52.2, 62.3, 103.8, 110.5, 115.6 (d, *J* = 21.6 Hz), 130.1 (d, *J* = 7.7 Hz), 147.8, 148.9, 158.5, 162.4 (d, *J* = 247.7 Hz); MS-ESI (*m*/*z*) 383.1 [M + H]^+^, 381.1 [M − H]^−^; HPLC: t_R_ = 6.53 min (100% purity).

### 3.2. HLM Stability Test 

Pooled human liver microsomes (adult male & female) were purchased from Merck (Schnelldorf, Germany). These microsomes were characterized in protein and CYP content. All incubations (final total volume 1050 µL) were made in the presence of an NADPH-regenerating system, consisting of 5 mM Glucose-6-phosphate, 5 U/mL Glucose-6-phosphate dehydrogenase and 1 mM NADP^+^. The substrate (100 μM), the NADPH regenerating system and 4 mM MgCl_2_ × 6 H_2_O in 0.1 M Tris buffer (pH 7.4 at 37 °C) were preincubated for 5 min in an incubator at 37 °C and 750 rpm. The incubation mix was split into 50 μL aliquots and the reaction was started by addition of the HLM. Thereby the microsomal protein content was standardized to 1 mg/mL. To follow the course of metabolism, the reaction tubes were quenched at selected time points (0, 10, 20, 30, 60, 120, 180 and 240 min) by adding 100 μL internal standard at a concentration of 22.5 µM in MeCN. The samples were vortexed for 30 s and centrifuged (19,800 relative centrifugal force/4 °C/10 min). The supernatant was directly used for LC-MS analysis (for detailed LC-MS conditions, see supplementary materials). All incubations were conducted in triplicates and incubations with heat-inactivated HLM were used to prove that analyte reduction results from metabolic degradation only. In all incubations, a limit of 1% organic solvent was not exceeded.

### 3.3. CYP Inhibition Test 

CYP inhibition (fluorimetric detection) assay was performed by Eurofins Panlabs Inc. (St. Charles, MO, USA) with human recombinant CYP enzyme and the appropriate CYP substrate. 

## 4. Conclusions

Replacement of the sulfur atom present in both known p38α MAP kinase inhibitors **ML3403** and **LN950** by a methylene group results in 2-alkylimidazole derivatives **1** and **2**, showing remarkably improved microsomal stability. Both compounds undergo minimal hydroxylation and *N*-dealkylation upon incubation with HLM. Trisubstituted imidazoles **1** and **2** display a 4-fold increase in binding affinity toward the kinase and possess a similar inhibition profile of p38α MAP kinase and the LPS-stimulated TNF-α release from HWB like their 2-alkylsulfanylimidazole counterparts **ML3403** and **LN950**. However, the exchange of the sulfur atom present in **ML3403** and **LN950** by a methylene group is accompanied with a slightly higher CYP inhibition profile of **1** and **2**. The most potent inhibitor, 2-alkylimidazole **2**, inhibits both the p38α MAP kinase as well as the LPS-stimulated TNF-α release from human whole blood in the low double-digit nanomolar range. Moreover, the excellent metabolic profile of **2** gives advantage over the mixed pharmacokinetics of the p38α MAP kinase reference inhibitor **ML3403** and its active metabolite. Therefore, 2-alkylimidazole **2** is a good alternative to evaluate the role of this kinase in in vitro and in vivo studies.

## Figures and Tables

**Table 1:** Evaluation of trisubstituted imidazoles for their inhibition of p38α MAP kinase (ELISA assay), their binding affinity to p38α MAP kinase (FP assay) as well as for their inhibition of TNF-α release in LPS-stimulated human whole blood (HWB assay).

Cpd	ELISA Assay p38α IC_50_ [nM] ^a^	FP Assay p38α K_i_ [nM] ^a^	HWB Assay TNF-α IC_50_ [nM] ^b^
**ML3403**	40 ± 5 ^c^	38 ± 1 ^e^	2979 ± 874
**1**	25 ± 2	11 ± 3	2539 ± 20
**LN950**	11 ± 0.9 ^d^	4 ± 1 ^e^	37 ± 4 ^d^
**2**	11 ± 5	1 ± 0.2	32 ± 1

^a^ n = 3; ^b^ n = 2; ^c^ value taken from Laufer and coworkers [7]; ^d^ value taken from Koch and coworkers [11]; ^e^ value taken from Ansideri et al. [16].

**Table 2:** (%) inhibition of the most important CYP isoenzymes at 10 µM.

CYP	1A2	2C9	2C19	2D6	3A4
**ML3403 ^a^**	66.7	79.0	83.5	31.1	79.2
**1**	85.6	85.1	71.0	90.2	97.6
**LN950**	28.0	91.5	63.0	41.5	61.0
**2**	69.4	61.9	83.6	78.8	92.1

^a^ values taken from Laufer and coworkers [6].

## Supplementary Materials

The following are available online, Figure S1 (Structure of **VX-745**) and Tables S1–S17 (metabolic stability of the title compounds in HLM and metabolite formation).
