# Supplementary material for: From 2-Alkylsulfanylimidazoles to 2-Alkylimidazoles: An Approach towards Metabolically More Stable p38α MAP Kinase Inhibitors
Source: Molecules. 2017 Oct 14;22(10):1729. doi: 10.3390/molecules22101729 (PMC6151569; doi:10.3390/molecules22101729)
Supplement: Supplementary file 1 [file molecules-22-01729-s001.pdf]

Supplementary Materials

From 2-alkylsulfanylimidazoles to 2-alkylimidazoles: An approach towards metabolically more stable p38α MAP kinase inhibitors

Fabian Heider[1], Urs Haun[1], Eva Döring[1], Mark Kudolo[1], Catharina Sessler[1], Wolfgang Albrecht[2], Stefan Laufer[1] and Pierre Koch[1,*]

[1]Department of Pharmaceutical and Medicinal Chemistry, Institute of Pharmaceutical Sciences, Eberhard Karls Universität Tübingen, Auf der Morgenstelle 8, 72076 Tübingen, Germany; pierre.koch@uni-tuebingen.de
[2]Teva-ratiopharm, Graf-Arco-Str. 3, 89079 Ulm, Germany

Table of Contents

Structure of **VX-745** (Figure S1) S2

Screening of metabolites by LC-MS analysis S2

Metabolic stability of **ML3403** in HLM (Table S1 – S5) S3

Metabolic stability of **1** in HLM (Table S6 – S10) S5

Metabolic stability of **LN950** in HLM (Table S11 – S14) S7

Metabolic stability of **2** in HLM (Table S15 – S17) S9

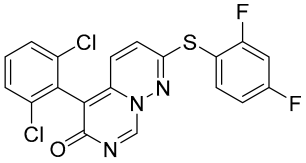

VX-745

Figure S1. Structure of selective p38α MAP kinase inhibitor **VX-745**.

Screening of Metabolites by LC-MS Analysis

Metabolite formation was analyzed with an Alliance 2695 Separations Module (Waters GmbH, Eschborn). Samples maintained at 4°C, the column temperature was set to 40°C and injection volume was 10 µL. The chromatographic separation for analytes **1** and **ML3404** was performed on a Phenomenex Synergi Max-RP column (150 x 4.6 mm; 5 µm); **LN950** and **2** on a Phenomenex Synergi Polar-RP column (150 x 4.6 mm; 5 µm) with a precolumn of the same material, respectively. An isocratic gradient of 8.5 min with 30% solvent A (90% H_2O, 10% ACN, 0.1% formic acid) and 70% solvent B (ACN, 0.1% formic acid) at a flow rate of 400 µL/min was used for **LN950**. **1** and **ML3404** were chromatographically separated by a binary gradient of 11.25 min with the equal solvents as mentioned before at a flow rate of 400 µL/min. The initial composition of 10% B was held for 20 sec, followed by a linear gradient up to 85% B in 5.8 min, holding for 30 sec, immediately changing to 10% B and reequilibrating at the end. The detection was performed on a Micromass Quattro micro triple quadrupole mass spectrometer (Waters GmbH, Eschborn) using the electrospray-ionization in the positive-mode. Correspondent to the analyte the spray voltage was set to 3.0-4.0 kV. The heated capillary operated at 250°C and the desolvation gas flow worked at 500 L/h.

Metabolic stability of ML3403 in HLM

Table S1. Degradation of **ML3404**

ML3403 [min]	#1 %	#2 %	#3 %	AVERAGE %	standard deviation
**0**	100.00	100.00	100.00	100.00	0.00
**10**	79.36	81.21	81.21	80.59	1.07
**20**	68.53	70.91	67.46	68.97	1.77
**30**	56.67	57.76	59.38	57.93	1.36
**60**	40.42	43.10	44.89	42.80	2.25
**120**	32.28	31.22	31.66	31.72	0.53
**180**	24.12	24.00	--*	24.06	0.08
**240**	15.55	15.71	17.47	16.25	1.07

Table S2. Formation of Metabolite **ML3603**: Sulfoxide of **ML3403** (*m/z* 421.5)

421.5 [min]	#1 %	#2 %	#3 %	AVERAGE %	standard deviation
**0**	0.60	0.67	0.62	0.63	0.04
**10**	15.61	14.30	15.67	15.19	0.78
**20**	25.79	26.25	24.86	25.63	0.71
**30**	38.75	37.61	35.26	37.21	1.78
**60**	52.92	52.65	53.40	52.99	0.38
**120**	74.60	79.32	66.69	73.54	6.38
**180**	73.27	70.16	--*	71.71	2.20
**240**	83.78	71.29	69.59	74.88	7.75

Table S3. Formation of Metabolite: Sulfone of **ML3403** (*m/z* 437.4)

437.4 [min]	#1 %	#2 %	#3 %	AVERAGE %	standard deviation
**0**	0.00	0.00	0.00	0.00	0.00
**10**	0.09	0.11	0.08	0.10	0.01
**20**	0.22	0.33	0.37	0.31	0.08
**30**	0.70	0.68	0.52	0.63	0.10
**60**	1.91	1.65	1.63	1.73	0.16
**120**	2.94	4.27	3.40	3.54	0.67
**180**	4.31	4.22	--*	4.27	0.06
**240**	6.80	4.89	5.65	5.78	0.96

Table S4. Formation of Metabolite: *N*-dealkylation of **ML3403** (*m/z* 301.4)

**301.4 [min]**	**#1 %**	**#2 %**	**#3 %**	**AVERAGE %**	**standard deviation**
**0**	0.00	0.01	0.00	0.00	0.01
**10**	0.93	0.87	0.80	0.87	0.06
**20**	1.00	1.50	1.27	1.26	0.25
**30**	1.95	1.90	1.87	1.91	0.04
**60**	2.85	2.66	2.60	2.70	0.13
**120**	3.09	3.15	2.90	3.05	0.13
**180**	2.75	2.58	--*	2.67	0.13
**240**	2.71	1.97	2.09	2.26	0.40

Table S5. Formation of Metabolite: *N*-dealkylation + sulfoxidation of **ML3403** (*m/z* 317.5)

**317.5 [min]**	**#1 %**	**#2 %**	**#3 %**	**AVERAGE %**	**standard deviation**
**0**	0.00	0.00	0.00	0.00	0.00
**10**	0.05	0.07	0.04	0.05	0.01
**20**	0.18	0.26	0.24	0.23	0.04
**30**	0.26	0.36	0.31	0.31	0.05
**60**	0.81	0.81	0.89	0.84	0.05
**120**	2.52	3.23	1.88	2.54	0.67
**180**	3.09	2.96	--*	3.03	0.09
**240**	5.74	2.97	3.15	3.95	1.55

*sample was unanalyzable

Metabolic stability of 2-alkylimidazole 1 in HLM

Table S6. Degradation of **1**

**1** [min]	#1 %	#2 %	#3 %	AVERAGE %	standard deviation
**0**	100.00	100.00	100.00	100.00	0.00
**10**	92.49	92.16	93.50	92.72	0.70
**20**	89.74	90.56	88.96	89.75	0.80
**30**	87.23	90.22	86.43	87.96	2.00
**60**	85.50	86.45	86.86	86.27	0.70
**120**	84.04	83.16	83.76	83.65	0.45
**180**	81.61	80.46	80.62	80.90	0.62
**240**	80.88	78.33	80.04	79.75	1.30

Table S7. Formation of Metabolite: *N*-dealkylation of **1** (*m*/*z* 283.6)

**283.6** [min]	#1 %	#2 %	#3 %	AVERAGE %	standard deviation
**0**	0.15	0.36	0.45	0.32	0.15
**10**	2.03	1.44	0.88	1.45	0.57
**20**	1.81	0.92	1.45	1.40	0.45
**30**	2.02	1.67	2.19	1.96	0.26
**60**	2.22	1.33	1.27	1.61	0.53
**120**	2.37	1.99	2.97	2.44	0.49
**180**	4.32	2.41	2.61	3.11	1.05
**240**	3.48	1.68	2.61	2.59	0.90

Table S8. Formation of Metabolite: oxidation (hydroxylation or *N*-oxide) of **1** [peak 1] (*m*/*z* 403.3)

**403.3 #1** [min]	#1 %	#2 %	#3 %	AVERAGE %	standard deviation
**0**	0.19	0.20	0.31	0.23	0.06
**10**	0.73	0.93	0.79	0.82	0.10
**20**	0.91	0.91	0.78	0.87	0.07
**30**	0.91	1.01	0.93	0.95	0.05
**60**	1.48	1.62	0.98	1.36	0.34
**120**	1.41	1.25	1.15	1.27	0.13
**180**	1.26	1.70	1.39	1.45	0.23
**240**	1.63	2.03	1.40	1.69	0.32

Table S9. Formation of Metabolite: oxidation (hydroxylation or *N*-oxide) of **1** [peak 2] (*m*/*z* 403.3)

403.3 #2 [min]	#1 %	#2 %	#3 %	AVERAGE %	standard deviation
0	0.66	0.67	0.57	0.63	0.06
10	1.66	2.06	2.06	1.93	0.23
20	1.79	2.80	1.67	2.08	0.62
30	2.07	2.47	1.85	2.13	0.32
60	2.07	2.90	1.81	2.26	0.57
120	2.40	3.84	2.14	2.80	0.92
180	2.89	2.96	2.26	2.70	0.38
240	2.67	3.92	2.53	3.04	0.77

Table S10. Formation of Metabolite: oxidation (hydroxylation or *N*-oxide) + *N*-dealkylation of **1** (*m*/*z* 299.5)

299.5 [min]	#1 %	#2 %	#3 %	AVERAGE %	standard deviation
0	1.53	1.55	1.67	1.58	0.08
10	3.66	4.22	3.66	3.85	0.32
20	4.07	4.86	3.91	4.28	0.51
30	3.82	5.28	4.48	4.53	0.73
60	5.28	7.05	4.53	5.62	1.30
120	5.37	7.77	4.59	5.91	1.66
180	6.13	6.54	5.66	6.11	0.44
240	6.11	9.20	5.66	6.99	1.93

Metabolic stability of LN950 in HLM

Table S11. Degradation of **LN950**

LN950 [min]	#1 %	#2 %	#3 %	AVERAGE %	standard deviation
**0**	100.00	100.00	100.00	100.00	0.00
**10**	87.14	86.54	86.92	86.86	0.31
**20**	78.90	77.41	75.93	77.42	1.48
**30**	63.72	63.03	59.15	61.96	2.47
**60**	54.65	53.40	47.91	51.99	3.59
**120**	40.46	37.14	34.09	37.23	3.19
**180**	32.27	34.26	29.76	32.10	2.25
**240**	30.74	29.49	25.25	28.49	2.88

Table S12. Formation of Metabolite: Sulfoxide of **LN950** (*m/z* 417.2)

417.2 [min]	#1 %	#2 %	#3 %	AVERAGE %	standard deviation
**0**	0.00	0.00	0.00	0.00	0.00
**10**	17.81	17.98	19.04	18.28	0.67
**20**	26.33	26.44	28.11	26.96	0.99
**30**	43.59	44.48	46.49	44.85	1.49
**60**	51.15	52.58	52.16	51.97	0.73
**120**	64.39	63.22	63.12	63.58	0.70
**180**	68.65	70.55	71.79	70.33	1.58
**240**	76.67	73.48	74.41	74.85	1.64

Table S13. Formation of Metabolite: Sulfone of **LN950** (*m/z* 433.2)

433.2 [min]	#1 %	#2 %	#3 %	AVERAGE %	standard deviation
**0**	0.00	0.00	0.00	0.00	0.00
**10**	0.00	0.16	0.16	0.11	0.09
**20**	0.00	0.43	0.45	0.29	0.25
**30**	1.09	1.16	1.45	1.24	0.19
**60**	1.77	1.97	2.27	2.00	0.25
**120**	3.27	4.09	3.96	3.77	0.44
**180**	4.33	4.91	5.65	4.96	0.67
**240**	5.33	5.19	6.27	5.59	0.59

Table S14. Formation of Metabolite: *N*-dealkylation of **LN950** (*m/z* 331.1)

**331.1 [min]**	**#1 %**	**#2 %**	**#3 %**	**AVERAGE %**	**standard deviation**
**0**	0.00	0.00	0.00	0.00	0.00
**10**	2.11	2.30	2.26	2.23	0.10
**20**	3.27	3.35	3.35	3.32	0.04
**30**	5.42	5.38	5.33	5.38	0.05
**60**	5.95	6.09	5.83	5.96	0.13
**120**	6.31	6.23	5.56	6.03	0.41
**180**	6.21	6.20	6.03	6.15	0.10
**240**	6.11	6.11	5.58	5.93	0.30

Metabolic stability of 2-alkylimidazole 2 in HLM

Table S15. Degradation of **2**

2 [min]	#1 %	#2 %	#3 %	AVERAGE %	standard deviation
0	100.00	100.00	100.00	100.00	0.00
10	95.05	97.48	98.92	97.15	1.96
20	91.81	94.65	94.25	93.57	1.54
30	91.71	89.70	94.12	91.84	2.21
60	91.28	88.75	95.94	91.99	3.65
120	91.51	89.68	93.50	91.57	1.91
180	90.53	86.95	93.80	90.42	3.43
240	89.68	87.61	91.90	89.73	2.15

Table S16. Formation of Metabolite: *N*-dealkylation of **2** (*m/z* 313.3)

313.1 [min]	#1 %	#2 %	#3 %	AVERAGE %	standard deviation
0	0.00	0.00	0.00	0.00	0.00
10	1.36	1.84	1.51	1.57	0.25
20	1.46	1.51	1.68	1.55	0.12
30	1.68	1.64	1.89	1.73	0.13
60	2.46	2.01	2.38	2.28	0.24
120	3.00	2.34	2.59	2.65	0.33
180	3.17	2.74	3.18	3.03	0.25
240	3.73	3.03	3.50	3.42	0.36

Table S17. Formation of Metabolite: oxidation (hydroxylation or *N*-oxide) + *N*-dealkylation of **2** (*m/z* 399.2)

399.2 [min]	#1 %	#2 %	#3 %	AVERAGE %	standard deviation
0	0.00	0.00	0.00	0.00	0.00
10	1.81	2.39	1.91	2.04	0.31
20	2.25	2.20	2.27	2.24	0.04
30	2.50	2.41	2.47	2.46	0.04
60	3.12	2.91	3.36	3.13	0.22
120	3.93	3.76	3.76	3.81	0.10
180	4.11	3.73	4.17	4.00	0.24
240	5.47	4.42	4.92	4.94	0.53
